# Intronic Parent-of-Origin Dependent Differential Methylation at the *Actn1* Gene Is Conserved in Rodents but Is Not Associated with Imprinted Expression

**DOI:** 10.1371/journal.pone.0048936

**Published:** 2012-11-08

**Authors:** John D. Calaway, José Ignacio Domínguez, Megan E. Hanson, Ezequiel C. Cambranis, Fernando Pardo-Manuel de Villena, Elena de la Casa-Esperon

**Affiliations:** 1 Curriculum in Genetics and Molecular Biology, Department of Genetics, Lineberger Comprehensive Cancer Center and Carolina Center for Genome Sciences of the University of North Carolina (UNC), Chapel Hill, North Carolina, United States of America; 2 Regional Center for Biomedical Research (C.R.I.B.), University of Castilla-La Mancha, Albacete, Spain; 3 Department of Biology, University of Texas Arlington, Arlington, Texas, United States of America; 4 Albacete Science and Technology Park, Albacete, Spain; VU University Medical Center, The Netherlands

## Abstract

Parent-of-origin differential DNA methylation has been associated with regulation of the preferential expression of paternal or maternal alleles of imprinted genes. Based on this association, recent studies have searched for parent-of-origin dependent differentially methylated regions in order to identify new imprinted genes in their vicinity. In a previous genome-wide analysis of mouse brain DNA methylation, we found a novel differentially methylated region in a CpG island located in the last intron of the *alpha 1 Actinin (Actn1)* gene. In this region, preferential methylation of the maternal allele was observed; however, there were no reports of imprinted expression of *Actn1*. Therefore, we have tested if differential methylation of this region is common to other tissues and species and affects the expression of *Actn1*. We have found that *Actn1* differential methylation occurs in diverse mouse tissues. Moreover, it is also present in other murine rodents (rat), but not in the orthologous human region. In contrast, we have found no indication of an imprinted effect on gene expression of *Actn1* in mice: expression is always biallelic regardless of sex, tissue type, developmental stage or isoform. Therefore, we have identified a novel parent-of-origin dependent differentially methylated region that has no apparent association with imprinted expression of the closest genes. Our findings sound a cautionary note to genome-wide searches on the use of differentially methylated regions for the identification of imprinted genes and suggest that parent-of-origin dependent differential methylation might be conserved for functions other that the control of imprinted expression.

## Introduction

DNA methylation plays very dynamic and diverse roles in genome function and architecture. A large body of DNA methylation studies has been dedicated to its contribution to gene expression regulation and, therefore, has been focused on methylated sites in genic and regulatory sequences. But methylated cytosines are also found throughout the genome and the function of most of them –if any- is still unknown. While cytosines can be equally methylated in the two parentally-inherited copies, there are sites in which allele-specific methylation is observed. These differentially methylated cytosines can be clustered in differentially methylated regions (DMRs) and it is assumed that these DMRs have potential implications in allelic expression of nearby genes.

In some cases differential methylation depends on cis-controlling elements, present in one allele but not the other. In other cases, methylation is biased towards the maternal or the paternal copy. These parent-of-origin dependent DMRs have characteristically been associated to the regulation of mammalian imprinted gene expression, in which preferential transcription of the paternal or the maternal copy occurs. Imprinted genes have attracted a lot of interest, due to both their particular mode of expression and their important roles, especially in embryonic development and in brain [1], [2], [3]. To this date, 150 genes have been reported in mouse at http://www.har.mrc.ac.uk/research/genomic_imprinting/; however, both sequence-based predictions and large-scale expression analyses have proposed larger numbers [4], [5], [6], [7]. In the run for the identification of novel imprinted genes, DNA methylation analyses have proved to be useful for finding novel DMRs that have lead to the discovery of new imprinted genes in their vicinity [8], [9], [10], [11].

In a recent study of the mouse brain methylome, we have found that strain-specific DMRs (*i.e.,* DMRs caused by *cis* effects) are more common than parent-of-origin DMRs (Calaway et al., unpublished results); similar findings have been reported in humans [10], [12]. Among the parent-of-origin dependent DMRs identified in our study, we found that most of them were associated with previously known imprinted genes. We also identified a novel DMR located in the last intron of the *alpha 1 Actinin (Actn1)* gene. This gene codes for the α-Actinin-1 microfilament protein that interacts dynamically with Actin. *Actn1* has not been previously reported as imprinted. Given the functional relevance of both DNA methylation and imprinted genes, we have focused the present study in the characterization of this new *Actn1* DMR. We have interrogated if differential methylation also occurs in other tissues, developmental stages and species. We have also explored the allelic expression of this imprinting candidate. We find no indication of imprinted expression at any of the tissues and developmental stages analyzed, although most of them show preferential maternal methylation at the DMR. Interestingly, we do find conservation of the *Actn1* DMR in rats but not in humans, suggesting that it may play a relevant functional role in murine rodents.

## Methods

### Mouse Lines and Samples

Two mouse strains were obtained from the Jackson Laboratory: 129S1/SvImJ (abbreviated 129S1) and PWK/PhJ (abbreviated PWK). For MS-RFLP and expression analyses, we collected whole-brain, kidneys, spleen, liver, testes, femoral muscle, and tail from two female and two male (129S1×PWK)F1 mice, as well as two female and two male (PWK×129S1)F1 mice at 6-weeks of age. In all crosses, dams are listed first and sires last. Additionally, we isolated whole brain and liver from a 45-day-old, male Sprague Dawley rat (Harlan). Dissected tissues were immediately frozen in liquid nitrogen and DNA and RNA were extracted according to standard procedures. Expression studies were also performed in RNA extracted from pooled E9.5 whole embryos and from E9.5 placentas: two female and two male (129S1×PWK)F1 pools and two female and two male (PWK×129S1)F1 pools. All procedures were conducted in accordance with NIH guidelines for the care and use of experimental animals and based on protocols approved by the Institutional Animal Care and Use Committee of UNC-Chapel Hill. Human hepatocytes, harvested from subjects with various causes of death, were purchased from ADMET Technologies, Inc. (Durham, NC, USA).

### Methylation-Sensitive Restriction Fragment Length Polymorphism (MS-RFLP) Analysis

**Figure 1:**
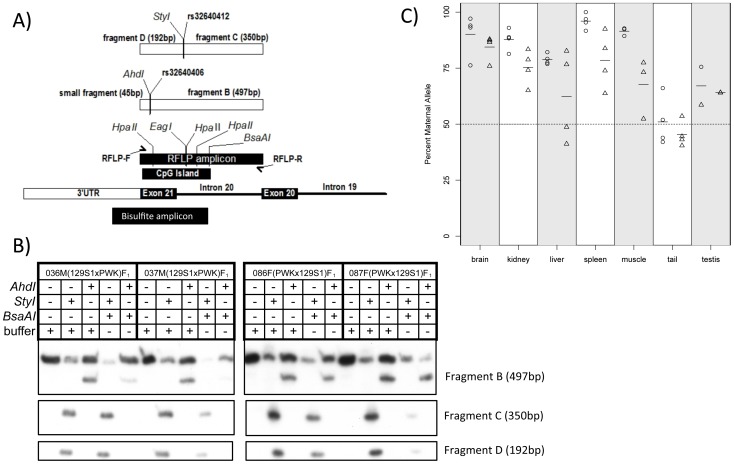
Maternal methylation of a novel DMR at the *Actn1* gene in diverse mouse tissues. A) A detailed map of the novel maternal *Actn*1 DMR is shown in the lower part. The diagram directly above shows the design for the MS-RFLP and bisulfite sequencing validation assays. Also included in this diagram are the locations of the methylation-sensitive enzyme restriction sites tested with MS-RFLP (*BsaAI, EagI* and *HpaII)*, the strain-specific cut sites (*AhdI* (present in 129S1 but not in PWK, due to SNP rs32640406) and *StyI* (present in PWK but not in 129S1, due to SNP rs32640412)), and the strain-specific resulting restriction fragments (see Methods). B) MS-RFLP results of four mouse liver samples. The matrix above the gel shows the different conditions for each individual lane. The plus sign (+) indicates addition, while the minus sign (−) indicated no addition of each corresponding endonuclease. C) Percent maternal methylation of an individual CpG (targeted by the *BsaAI* endonuclease) within different tissues. Circles represent individual (PWK×129S1)F_1_ mice, while triangles represent individual (129S1×PWK)F_1_ mice. Horizontal bars represent percent maternal methylation averages.

Genomic DNA was first digested with *EcoRI* (New England Biolabs) to reduce structural complexity and ensure that the restriction site is accessible to subsequent endonucleases digestions [13]. Samples were then either digested with methylation-sensitive enzymes *BsaAI, EagI, HpaII* (NEB), or mock treated (buffer only). The cut sites of *BsaAI, EagI* and *HpaII* include one or more of the CpGs targeted for PCR amplification. Methylation-sensitive digested samples were then PCR amplified using a RFLP forward primer and a RFLP reverse primer, and radiolabeled dCTP (Figure 1A and Table S1). PCR products were digested with either 129S1-specific *StyI,* PWK-specific *AhdI,* or mock treated. Samples were electrophoresed through 5% acrylamide denaturing gel and visualized by X-ray film.

For allelic ratio quantitation, X-ray films were scanned (Epson) and the Tiff images were imported into ImageJ [14] for densitometry. We arbitrarily named the undigested RFLP amplicon, “A” (542 bp); the fragment generated by *AhdI* digestion, “B” (497 bp); the larger fragment from *StyI* digestion, “C” (350 bp); and the smaller *StyI* fragment, “D” (192 bp) (Figure 1A). The relative amount of each parental allele was determined by the ratio of the sum of the absolute density of allele-specific fragments (Figure S1A) and to the total absolute density of all bands:

*StyI* digestion:methylated PWK allele = (C+D)/(A+C+D) (direct measurement)

*AhdI* digestion: methylated 129S1 allele = B/(A+B) (direct measurement)

This method for calculating percent methylated parental alleles gave an inflationary result for PWK and a deflationary result for 129S1 based on buffer-only controls. We, therefore, created a panel with diverse ratios of PWK and 129S1 genomic DNA and digested with *StyI* or *AhdI* to serve as a standard curve (PWK/129S1∶0/100, 5/95, 25/75, 50/50, 75/25, 95/5, 100/0). This allowed us to interpolate “actual” PWK/129S1 allelic ratios from “observed” ratios (Figure S1B). We normalized all RFLP densitometry measurements by applying the respective interpolation equations.

We utilized the R environment for conducting the two-factor ANOVA and t-tests for determining significant differences in maternal methylation between tissues and reciprocal crosses.

### Sodium Bisulfite Sequencing

One microgram of genomic DNA from mouse (n = 2), rat (n = 1) or human (n = 1) tissues was treated with Zymo Research EZ DNA Methylation-Gold™ Kit according to the manufacturer’s protocol. Species-specific primers were designed to flank and amplify the bisulfite converted DMR (Table S1). Purified PCR products were cloned and sequenced. The false discovery rate for methylated CpG’s was calculated by the number of unconverted non-CpG cytosines divided by the total number of non-CpG cytosines across individual PCR reactions.

### Expression Analysis

**Figure 2:**
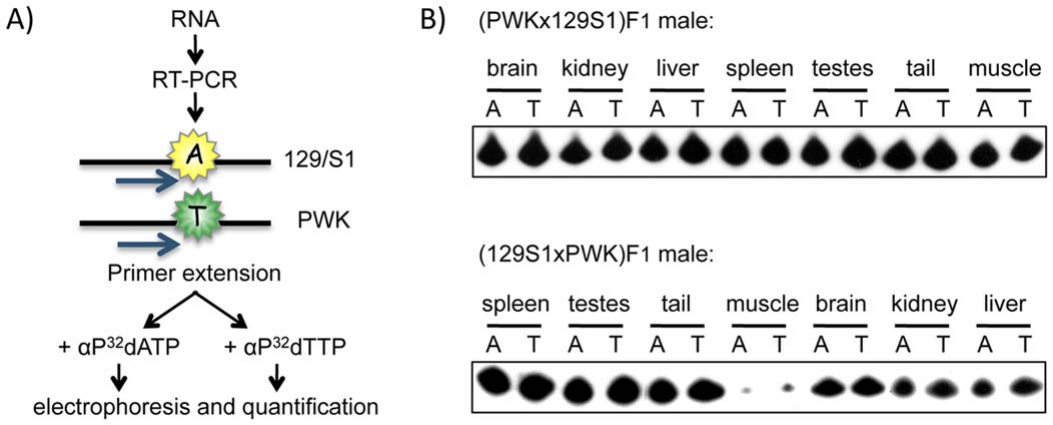
*Actn1* allelic expression analysis by SNuPE. A) Summary of the SNuPE (Single Nucleotide Primer Extension) method. B) Autoradiogram of SNuPE products after electrophoresis, showing biallelic expression of *Actn1* in all tissues analyzed.

**Figure 3:**
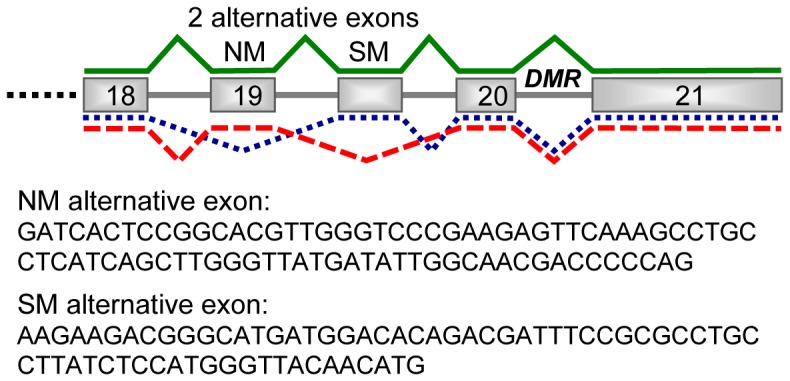
Mouse *Actn1* isoforms. They result from alternative splicing of two exons at the 3′ end of the gene. These exons are designated SM (smooth muscle) and NM (non-muscular) due to their homology to previously described rat alternative exons (Kremerskothen et al. 2002). Exons are numerated 18–21 as on Ensembl transcript isoform ENSMUST00000021554. The position of the DMR in is indicated in the last intron (image not drawn at scale).

Allele-specific expression of *Actn1* was analyzed by two independent methods: sequencing or Single Nucleotide Primer Extension (SNuPE) analysis of SNPs present in RT-PCR products. RNA of the above described mouse tissue samples was retrotranscribed (using *Actn1-*specific primers), followed by PCR (Tables S1 and S2), using the appropriate controls to avoid genomic DNA amplification. An informative SNP at position 12∶81,269,902 (m37) was selected for analysis of the relative expression of alleles by Single Nucleotide Primer Extension (SNuPE) [15], [16] (Figure 2). Sanger sequencing of the same RT-PCR products was performed in order to verify the SNuPE results; *Actn1* allelic expression was determined by chromatogram inspection of three SNPs at positions 12∶81,269,902, 12∶81,269,896 and 12∶81,269,456 (m37) (Table S2 and Figure S2B). As additional confirmation, we performed RT-PCR of brain samples with a different set of primers (Tables S1 and S2). The resulting products were subjected to Sanger-sequencing to test for allelic expression at SNPs located in positions 12∶81,284,503 and 12∶81,274,013 (m37) (Figure S2B). Allelic expression analysis of *Actn1* isoforms was also performed by sequencing of RT-PCR products, using isoform-specific primers Actn1-18SM, Actn1-NM20 and Actn1-NMSM (Figure 3 and Tables S1 and S2).

Allelic expression of *Zfp36l1, AK037382* and *Dcaf5 (Wdr22)* in embryonic and adult mouse tissues was determined by RT-PCR (see primers in Table S1), followed by Sanger-sequencing and chromatogram inspection of SNPs between 129S1 and PWK alleles described at http://www.sanger.ac.uk/cgi-bin/modelorgs/mousegenomes/snps.pl.

## Results

### A Novel *Actn1* DMR has Preferential Maternal Methylation in Diverse Mouse Tissues

In a previous study, we performed a genome-wide methylation study of the mouse brain DNA by methylation-sensitive single nucleotide polymorphism (MSNP) analysis (Calaway et al., unpublished results). This analysis was applied to brain DNAs of F1 offspring of reciprocal crosses between 129S1 and PWK mice. Our study identified a novel parent-of-origin dependent DMR associated with two SNPs, rs32640412 and rs32641208, located in a CpG island and in the last intron of the *Actn1* gene (Figure 1A). Maternal-specific methylation of this DMR was confirmed by methylation-sensitive restriction fragment length polymorphism (MS-RFLP) analysis (Calaway et al., unpublished results).

In this study, we have expanded the methylation analysis of the *Actn1* gene. First, we examined whether the *Actn1* DMR occurs in tissues other than brain. Genomic DNA isolated from whole brain, kidney, liver, spleen, testis, tail, and femoral muscle from four (PWK×129S1)F1 mice and four (129S1×PWK)F1 mice were subjected to MS-RFLP. In this technique, restriction digestion with methylation-sensitive endonucleases is performed prior to PCR amplification of the region under our study; consequently, only methylated restriction sites are preserved and, thus, amplified. In order to determine the methylation status of each allele, an additional digestion was performed after PCR and before electrophoresis with strain-specific endonucleases *StyI* (which only digests the PWK allele) or *AhdI* (specific for the 129S1 allele) (Figure 1A). Depending on the direction of the cross, the percent methylated maternal allele or paternal allele was calculated by the ratio of relative fragment densities of either *StyI* (PWK) or *AhdI* (129S1) digestions (see Materials and Methods). Both methylation measurements were correlated for each of the three methylation-sensitive enzymes used: *BsaAI, EagI* and *HpaII* (Figure S3). Examples of *BsaA*I MS-RFLP results for liver are shown in Figure 1B. Figure 1C represents the percent maternal methylation at a single CpG internal to the *BsaAI* cut site (chr12∶81,269,613 (m37), Figure 1A) in diverse tissues. We observed that differential methylation at *Actn1* is not unique to brain. Similar results were obtained for both *EagI* and *HpaII* digestions (Figure S4).

Moreover, we observed differences in the mean percent maternal methylation at the *BsaAI* CpG site between tissue types (Figure 1C). Pairwise t-tests revealed significant differences in the percent maternal methylation between tail and other tissues (α <0.05 in both types of F1 mice, Table S3). In addition, a two factor ANOVA test identified statistically significant differences not only between tissue types (F = 16.733, p-value = 7.639•10^−10^), but also between reciprocal F1 hybrids (F = 20.413, p-value = 4.821•10^−5^). However, the varying degree of maternal methylation between tissues is not significantly different between reciprocal F1s.

### *Actn1* DMR Extent and Conservation in Murine Rodents

To determine if the *Actn1* DMR is unique to mice or, on the contrary, conserved in other mammalian species, we analyzed the orthologous regions in humans and rats. Located distally on chromosome 12 in mouse (81,268,534-81,361,303, NCBI37/mm9), *Actn1* is orthologous with a region on rat chromosome 6 (103,187,905–103,282,948, Baylor 3.4/rn4) and human chromosome 14 (69,341,075–69,359,000, GRCh37/hg19). We predicted the location of the human and rat orthologous DMRs based on the assumption that they are typically associated with regions of high CpG dinucleotide density (CpG islands) and their shores [17], [18]. We used the following criteria to define a CpG island: a GC content greater than 50% and an observed/expected (O/E) CpG ratio greater than 0.6 over a 200 bp minimum length. Both the mouse *Actn1* CpG island (27CpGs, 57.3% GC content over 302 bp, CpG O/E 1.10) and the rat *Actn1* CpG island (25CpG, 60.8% GC content over 265 bp, CpG O/E 1.03) span most of the last exon coding region and part of the last intron (intron 20 in reference sequences NM_134156.2 for mouse and NM_031005.3 for rat) (Figure 1A). In humans, the CpG island is larger (40 CpGs, 68.2% GC content, length 393 bp, CpG O/E 0.91) and includes a large portion of the 3′UTR (reference sequence NM_001102.3).

**Figure 4:**
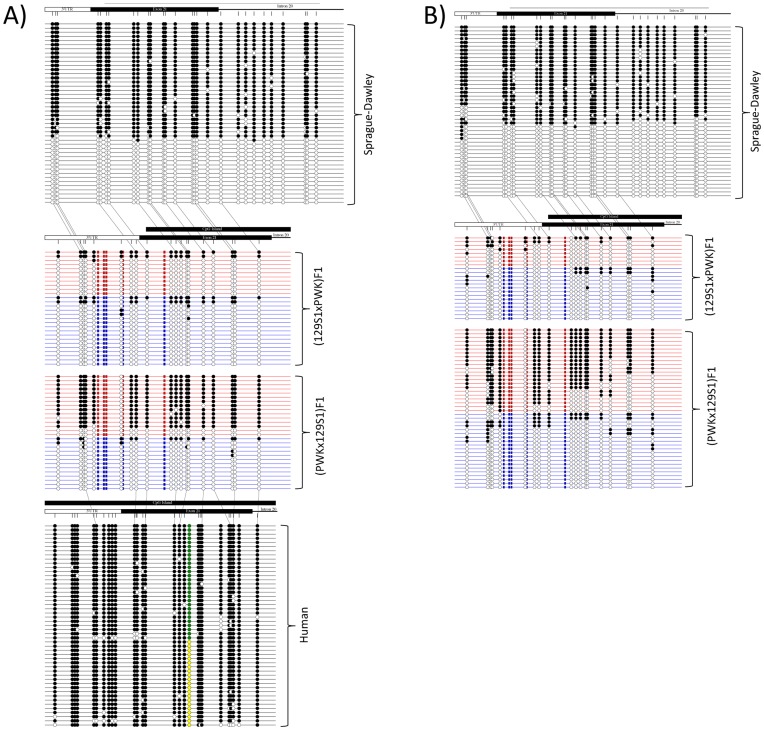
Bisulfite sequencing analysis of the *Actn1* DMR in mouse, rat and human tissues. Panel A shows bisulfite sequencing results from clones isolated from rat liver, mouse liver, and human hepatocytes. Each horizontal line represents a unique clone. Red and blue lines represent maternal and paternal parent-of-origin, respectively, based on five strain-specific variants. Open circles are unmethylated CpGs, while closed circles are methylated CpGs. Green and yellow circles shown in human hepatocyte clones represent variant rs11557769 and distinguish parental alleles, although parent-of-origin is unknown. Orthologous CpGs are connected by dotted lines (in relation to mouse). Panel B shows bisulfite sequencing results from clones isolated from rat right brain hemisphere (top) and mouse right brain hemispheres (bottom).

We investigated the methylation status of multiple CpG sites at the *Actn1* CpG islands of these three species by sodium bisulfite treatment followed by PCR and sequencing analysis. (PWK×129S1)F1 mice displayed brain maternal hypermethylation and paternal hypomethylation, while (129S1×PWK)F1 mice showed weak maternal methylation and sporadic paternal methylation across the 19 CpG’s sequenced (Figure 4B). We found similar results in mouse liver DNA (Figure 4A). These data indicate that methylation at the *Actn1* DMR depends both on the parental and the strain origin (the sequences in *cis*) of the CpG sites. They are also consistent with the MS-RFLP results of (129S1×PWK)F1 mice (Figure 1C), which showed more methylation variability and, on average, lower percent of maternal methylation than (PWK×129S1)F1 animals. In rat, we were unable to identify a polymorphism for establishing a parent-of-origin anchor within the 347 bp bisulfite amplicon, due to the limited genetic diversity between available rat strains. Nevertheless, we observed a strongly polarized population of hypermethylated or hypomethylated bisulfited amplicons suggestive of differential methylation in both rat brain and liver DNA (Figures 4A and 4B). In contrast, the human *ACTN1* DMR is consistently methylated at greater than 94% (false discovery rate of 0.68%) in hepatocytes (Figure 4A). The presence of a T → A transversion (rs11557769, at position 69,341,653 (GRCh37/hg19)) allowed us to conclude that both the maternal and paternal alleles are hypermethylated (Figure 4A). Therefore, in human hepatocytes, the orthologous region to the mouse *Actn1* DMR is not differentially methylated, while biased methylation is conserved in murine rodents.

We also examined the methylation upstream and downstream of the mouse *Actn1* DMR, by performing bisulfite treatment of liver DNA followed by PCR amplification of flanking sequences. Our assay design was constrained by the scarcity of informative SNPs between 129S1 and PWK and the profusion of homopolymers in the sequences surrounding the DMR. Nevertheless, we generated data for one region upstream (81,270,081-81,270,495, NCBI37/mm9) and one region downstream (81,263,196-81,263,520, NCBI37/mm9) from our previous DMR bisulfite assay (Table S1 and Figure S5). Comparative analysis of the results of the reciprocal crosses shows that the preferential maternal methylation observed in the DMR does not extend to these neighboring regions (Figure S5). Therefore, in mouse liver DNA, the DMR appears to be confined to the vicinity of the last *Actn1* intron.

### Expression Studies of *Actn1* do not Reveal Imprinting Effects

Next, we tested if the *Actn1* parent-of-origin dependent DMR is associated with imprinted expression of nearby genes. To date, there are no reports of imprinted expression of *Actn1*. To investigate if such is the case, we analyzed the expression of the mouse gene in RNA obtained from the same tissues and F1 individuals studied for DNA methylation purposes. In order to distinguish maternal from paternal expression, we sequenced the *Actn1* coding sequences and identified several SNPs between the 129S1 and PWK strains. The relative expression of 129S1 and PWK alleles was tested by Single Nucleotide Primer Extension (SNuPE) at a SNP located in chr12∶81269902 (m37) (Figure 2A) [15], [16]. In spite of the presence of the *Actn1* DMR, we always observed *Actn1* biallelic expression, finding no indication of allelic expression bias in any sex, F1 or tissue type (Figure 2B and Figure S2A). These results were validated by direct sequencing of the cDNAs generated in the SNuPE analysis (Figure S2B). We also confirmed biallelic expression of *Actn1* at other SNPs (chr12∶81,284,503 and chr12∶81,274,013 (m37)) by independent RT-PCR and sequencing analyses of F1 RNA samples (Table S2 and Figure S2B).

Imprinted gene expression can be restricted to specific isoforms or developmental stages, being particularly common in placenta and embryonic tissues [19], [20], [21], [22], [23]. In order to test if a DMR effect on *Actn1* transcription is restricted to prenatal stages, allelic expression analysis by RT-PCR and sequencing was applied to (129S1×PWK)F1 and (PWK×129S1)F1 E9.5 embryos and placentas of both sexes (Table S2). The results of this analysis showed no apparent allelic expression bias. We also tested if the DMR had an imprinted expression effect restricted to any specific *Actn1* isoform. In rat, three isoforms resulting from two alternatively spliced exons (NM (“non-muscle”) and SM (“smooth muscle”) exons) have been described of this gene [24]. We found these three *Actn1* isoforms are also present in mouse (Figure 3). Sequencing analysis of RT-PCR products with isoform-specific primers (Table S2) revealed that expression of the three isoforms is biallelic in (129S1×PWK)F1 and (PWK×129S1)F1 adult brain, E9.5 placentae and embryos of both sexes. Therefore, our results show that the *Actn1* parent-of-origin dependent DMR observed in F1 mice derived from PWK and 129S1 strains is not associated with *Actn1* imprinted expression in any of the sexes, tissues, developmental stages and isoforms analyzed.

## Discussion

During a genome-wide methylation study of the mouse brain DNA, we identified a novel parent-of-origin dependent DMR in the 3′ end of the *Actn1* gene (Calaway et al. unpublished results). We have confirmed that this intronic DMR is maternally methylated in brain of F1 individuals derived from reciprocal crosses between 129S1 and PWK strains by MS-RFLP and bisulfite analyses. We have extended our mouse study to a tissue panel that is representative of all three germ layers: ectoderm (brain), mesoderm (kidney, spleen, muscle and testes) and endoderm (liver). All examined tissues (except for the tail, a body part of mixed origin [25]) display preferential maternal methylation of the *Actn1* DMR. These results suggest that the imprint was established very early during embryogenesis. Although this imprint persists through subsequent differentiation, the extent of maternal methylation varies significantly among tissue types, as well as between reciprocal crosses. Differences in allelic methylation levels among tissues, as well as interindividual variation, have also been observed in other DMRs, such as those associated with several imprinted genes [26], [27], [28], [29].

Traditionally, parent-of-origin dependent DMRs have been identified due to their proximity to imprinted genes. In fact, they have been found even within imprinted gene sequences (*e.g.,* introns). Therefore, we examined the expression of *Actn1* in the same tissue panel as the methylation analyses. We found no indication of allelic imbalance in any of the adult tissues examined. We also explored the possibility that imprinted expression could be restricted to particular isoforms or to specific developmental stages (particularly embryonic and extraembryonic tissues) [19], [20], [21]. We found three isoforms of mouse *Actn1* that result from alternative splicing of two alternative exons. Nevertheless, none of them showed allelic expression bias in adult brain, E9.5 embryos or E9.5 placentas of both reciprocal crosses and sexes. Therefore, our results do not support an association of parent-of-origin dependent methylation at *Actn1* with imprinted expression of the same gene. However, we cannot exclude the possibility that such imprinting could be restricted to a very specific cell type and/or developmental stage that have not been captured by our study.

We also tested if the DMR is involved in the imprinted expression of the next closest transcripts: *AK037382* and *Zfp36l1*, which are overlapping and close to the 3′ end of *Actn1*, respectively, as well as *Dcaf5 (Wdr22)*, a gene near to the 5′ end of *Actn1* (see Materials and Methods). However, we did not detect imprinted expression of these genes in any of the adult and embryonic mouse tissues analyzed (data not shown). In fact, the closest known imprinted genes are located as far as 29 Mb apart in the *Dlk1-Dio3* cluster (http://www.mousebook.org/catalog.php?catalog=imprinting).

From these results, we conclude that parent-of-origin dependent DMRs can be uncoupled from imprinted expression effects on nearby genes and, therefore, they are not perfect predictors of imprinted expression of genes located in their immediate proximity. This has important implications for large-scale searches for novel imprinted genes through the identification of parent-of-origin dependent epigenetic marks. In fact, recent genome-wide studies have also revealed the existence of novel parent-of-origin dependent DMRs outside known imprinted regions [8], [11], [12], [30]. Although deeper analyses have allowed the association of several of these DMRs with imprinted genes, the role of other DMRs remains unclear. Some are located within introns (as the *Actn1* DMR), while others are in intergenic regions and far from gene sequences [30].

We have gone a step further and interrogated if the *Actn1* DMR is an oddity unique to the mice used in our study (*i.e.,* intersubspecific hybrids [31]), or if it is also present in other species. We have found that, while orthologous *Actn1* CpG islands exist in other mammals, differential methylation is conserved in murine rodents (mouse and rat) but absent in humans. Our findings open an interesting question: can parent-of-origin dependent DMRs have been evolutionarily selected due to a functional role other than imprinted expression regulation? In other words: is the regulation of imprinted expression the only function of these DMRs? Several evidences indicate that DMRs and imprinted gene expression do not always go hand in hand. Within species, uncoupling of DMRs from imprinted expression can occur even in those typically associated with imprinted genes: for instance, paternal methylation of the imprinting control region of the *Rasgrf1* gene has been observed even in those tissues in which this gene is biallelically expressed [32]. This suggests that certain parent-of-origin dependent DMRs may have been selected for imprinting regulation and retained in all tissues throughout development, although imprinted expression would require tissue-specific factors in addition to differential methylation [1]. However, these selective pressures would be insufficient for the existence of other class of DMRs: those that are associated to imprinted expression in some species but not others. Such is the case of DMRs of the *IGF2R* gene, which is a gene that is imprinted in mice but not humans, while parent-of-origin differential methylation is present in both species [33], [34], [35]. Our finding adds an additional twist: DMR conservation in murine rodents in the absence of imprinted expression evidence.

A simple explanation for the *Actn1* DMR murine conservation is selection due to its necessary contribution to the regulation of chromosomal functions other than imprinted expression. In sexually reproducing organisms, parent-of-origin dependent epigenetic differences have been associated to phenomena as diverse as chromosome segregation or elimination and can affect replication, recombination and heterochromatinization of chromosomes in many sexually reproducing organisms [36], [37]. They have also been proposed to contribute to meiotic pairing and recombination and to DNA repair [36], [37]. From this broad perspective, large-scale studies of differentially methylated regions have the potential to unveil not only new imprinted genes, but also novel parent-of-origin dependent phenomena.

## Supporting Information

Figure S1 *Actn1* DMR analysis by RFLP. A) Sample gel displaying DNA fragments resulting from RFLP analysis of the *Actn1* DMR. The undigested amplicon is arbitrarily named fragment A (542 bp). *StyI* digestion of this amplicon yields fragments C (350 bp) and D (192 bp). *AhdI* digestion yields fragment B (497 bp). A smaller, 45 bp fragment is generated from the AhdI digestion but migrates with free αP^32^-dCTP and, therefore, was not included in the data analysis. B) Plot of artificially created PWK/129S1 allelic ratios for the analysis of MS-RFLP data of *Actn1* DMR. The X- and Y-axes are the fraction of expected and observed methylated parental alleles, respectively. Also shown are the polynomial interpolation equations used to normalize the observed allelic ratios.
(TIF)

Figure S2 Allelic expression analyses of *Actn1* in diverse mouse tissues shows biallelic expression. A) Results of SNuPE analyses of *Actn1* RNA of adult tissues of 2 females and 2 males of each cross, expressed as average proportion of 129S1 allele ± S.D. B) Examples of *Actn1* cDNA sequence analysis at two polymorphisms.
(TIF)

Figure S3 Correlation of maternal and paternal allelic methylation measurements at the *Actn1* DMR. Depending on the direction of the cross, the percent maternal methylation and the percent paternal methylation measurements are calculated by the ratios of *StyI* or *AhdI* restriction fragment densities. The direct measurements of maternal methylation are plotted against the direct measurements of paternal methylation for each individual methylation-sensitive endonuclease. Fitted line equations and R^2^ values are shown in the graph interior.
(TIF)

Figure S4 Percent maternal methylation of *Actn1* DMR based on *EagI* and *HpaII* MS-RFLP. Box and whisker plots showing the lower quartile, median, and upper quartile of percent maternal methylation by cross and by tissue type determined by *HpaII* or *EagI* MS-RFLP.
(TIF)

Figure S5 Bisulfite sequencing analysis of two regions flanking the *Actn1* DMR in mouse liver tissues. Panel A shows regions of preferential methylation investigated by bisulfite sequencing. Solid red lines represent sequenced regions, while dotted lines represent gaps in sequenced regions. Panel B shows a schematic representation of the positions and sizes of the regions selected for methylation analysis by bisulfite sequencing respect to the location of the last two exons of *Actn1* (exons 20 and 21, ENSMUSE00000114871 and ENSMUSE00000335764, respectively). Two regions, situated downstream (DNS BSP amplicon) and upstream (UPS BSP amplicon) of the region in which we observed differential methylation (BSP amplicon) (Figure 4), were selected for bisulfite sequencing analysis and the results are shown below the schematic. Each horizontal line represents a unique clone. Red and blue marks symbolize maternal and paternal alleles, respectively, of strain-specific variants. Open circles represent unmethylated CpGs, while closed circles are methylated CpGs.
(TIF)

Table S1 List of primers used in the MS_RFLP (RFLP-), Bisulfite-PCR (BSP-), RT-PCR and sequencing or SNuPE (Snu-) analyses.
(TIF)

Table S2 Summary of *Actn1* allelic expression analyses performed (see Supplemental Table S1 for primer’s sequences)
(TIF)

Table S3 Pairwise t-tests of percent maternal methylation at the *Actn1* DMR between tissues. Shown are the p-values (α<0.05)
(TIF)
