# Supplementary figures and images for: Intronic Parent-of-Origin Dependent Differential Methylation at the Actn1 Gene Is Conserved in Rodents but Is Not Associated with Imprinted Expression
Source: PLoS One. 2012 Nov 8;7(11):e48936. doi: 10.1371/journal.pone.0048936 (PMC3493592; doi:10.1371/journal.pone.0048936)

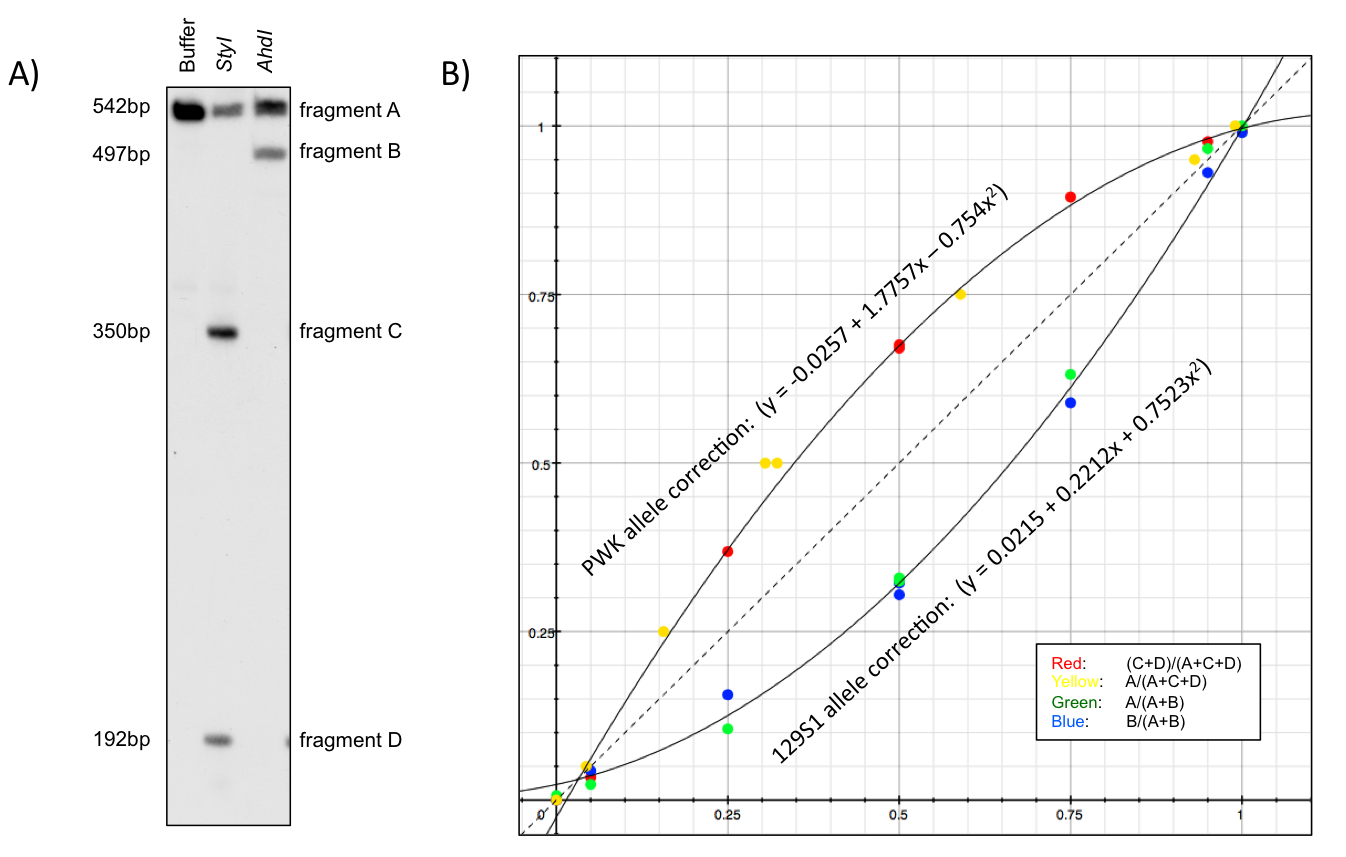

Supplement: Figure S1 — Actn1 DMR analysis by RFLP. A) Sample gel displaying DNA fragments resulting from RFLP analysis of the Actn1 DMR. The undigested amplicon is arbitrarily named fragment A (542 bp). StyI digestion of this amplicon yields fragments C (350 bp) and D (192 bp). AhdI digestion yields fragment B (497 bp). A smaller, 45 bp fragment is generated from the AhdI digestion but migrates with free αP32-dCTP and, therefore, was not included in the data analysis. B) Plot of artificially created PWK/129S1 allelic ratios for the analysis of MS-RFLP data of Actn1 DMR. The X- and Y-axes are the fraction of expected and observed methylated parental alleles, respectively. Also shown are the polynomial interpolation equations used to normalize the observed allelic ratios. (TIF) [file pone.0048936.s001.tif]

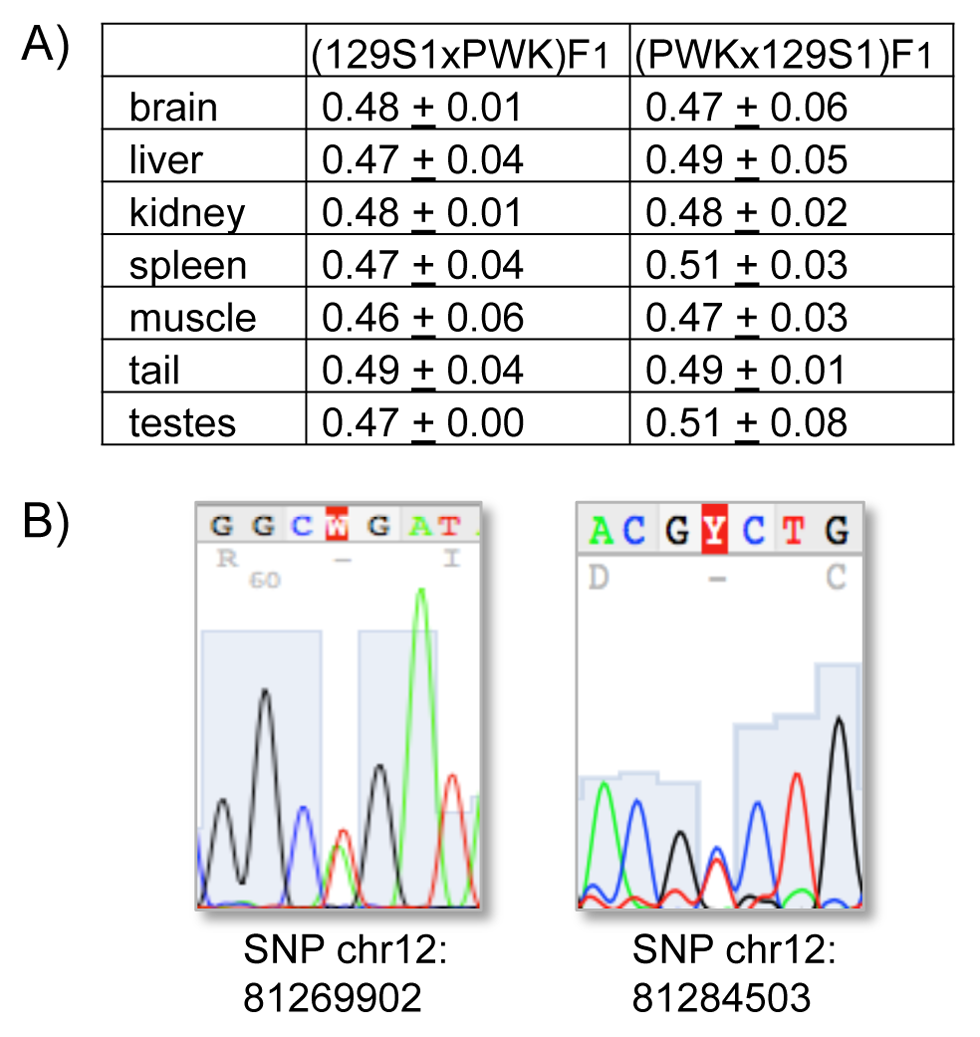

Supplement: Figure S2 — Allelic expression analyses of Actn1 in diverse mouse tissues shows biallelic expression. A) Results of SNuPE analyses of Actn1 RNA of adult tissues of 2 females and 2 males of each cross, expressed as average proportion of 129S1 allele ± S.D. B) Examples of Actn1 cDNA sequence analysis at two polymorphisms. (TIF) [file pone.0048936.s002.tif]

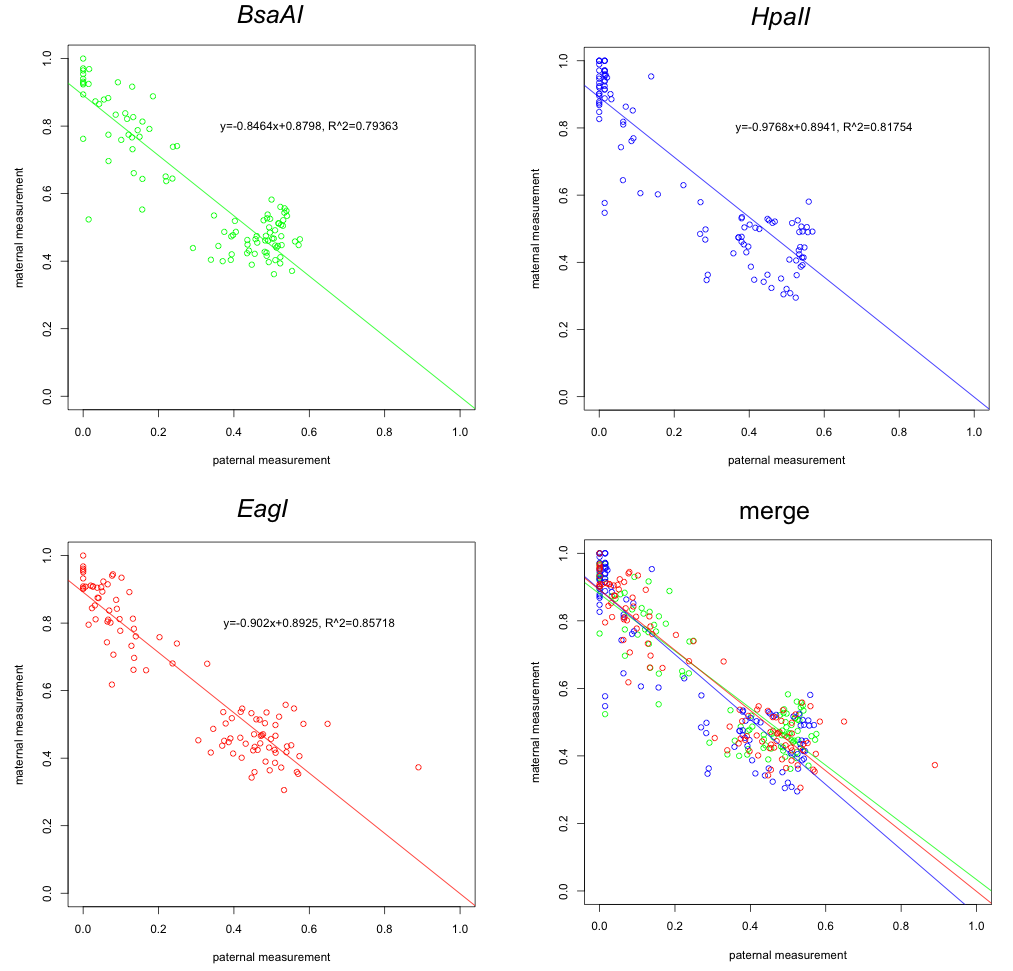

Supplement: Figure S3 — Correlation of maternal and paternal allelic methylation measurements at the Actn1 DMR. Depending on the direction of the cross, the percent maternal methylation and the percent paternal methylation measurements are calculated by the ratios of StyI or AhdI restriction fragment densities. The direct measurements of maternal methylation are plotted against the direct measurements of paternal methylation for each individual methylation-sensitive endonuclease. Fitted line equations and R2 values are shown in the graph interior. (TIF) [file pone.0048936.s003.tif]

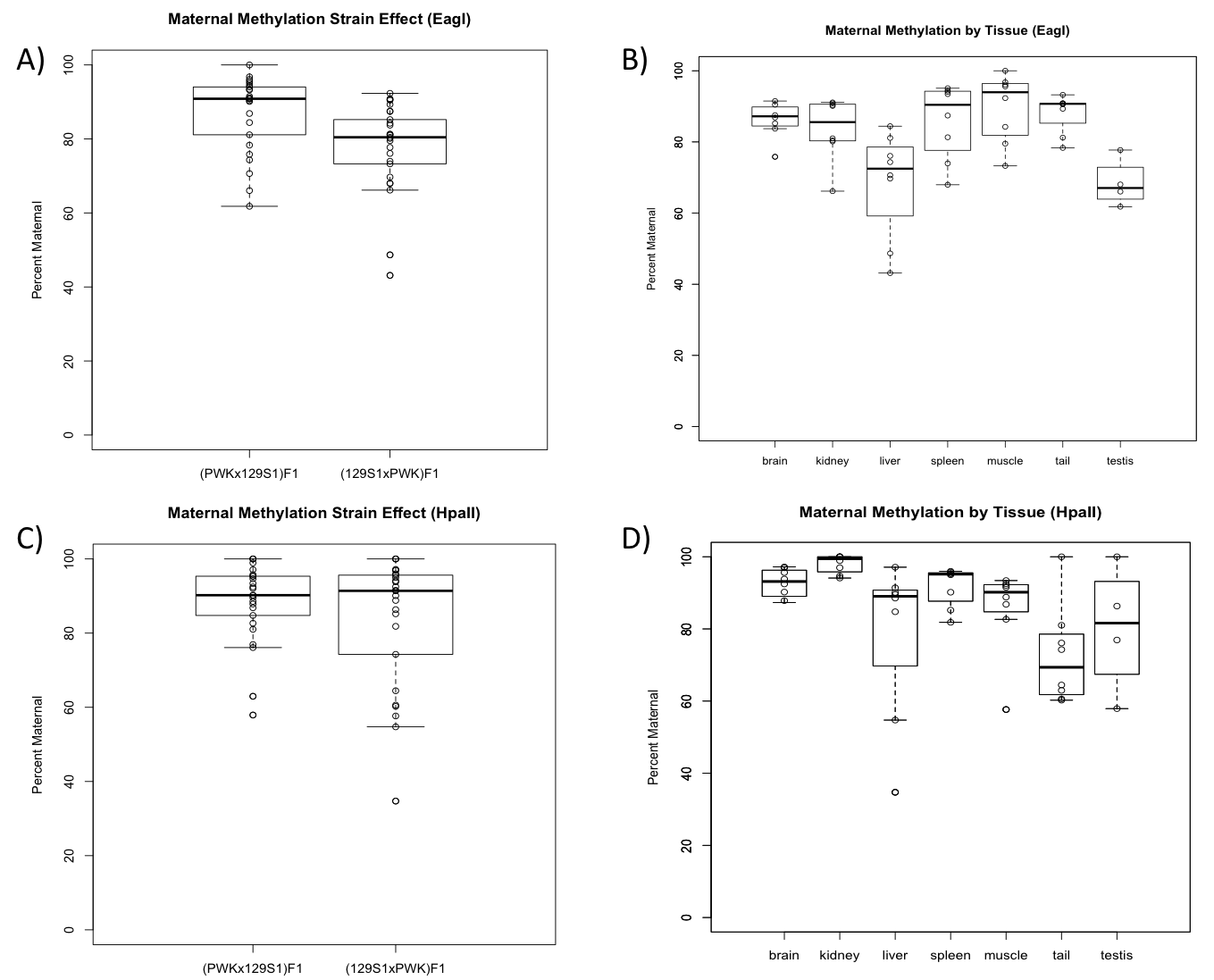

Supplement: Figure S4 — Percent maternal methylation of Actn1 DMR based on EagI and HpaII MS-RFLP. Box and whisker plots showing the lower quartile, median, and upper quartile of percent maternal methylation by cross and by tissue type determined by HpaII or EagI MS-RFLP. (TIF) [file pone.0048936.s004.tif]

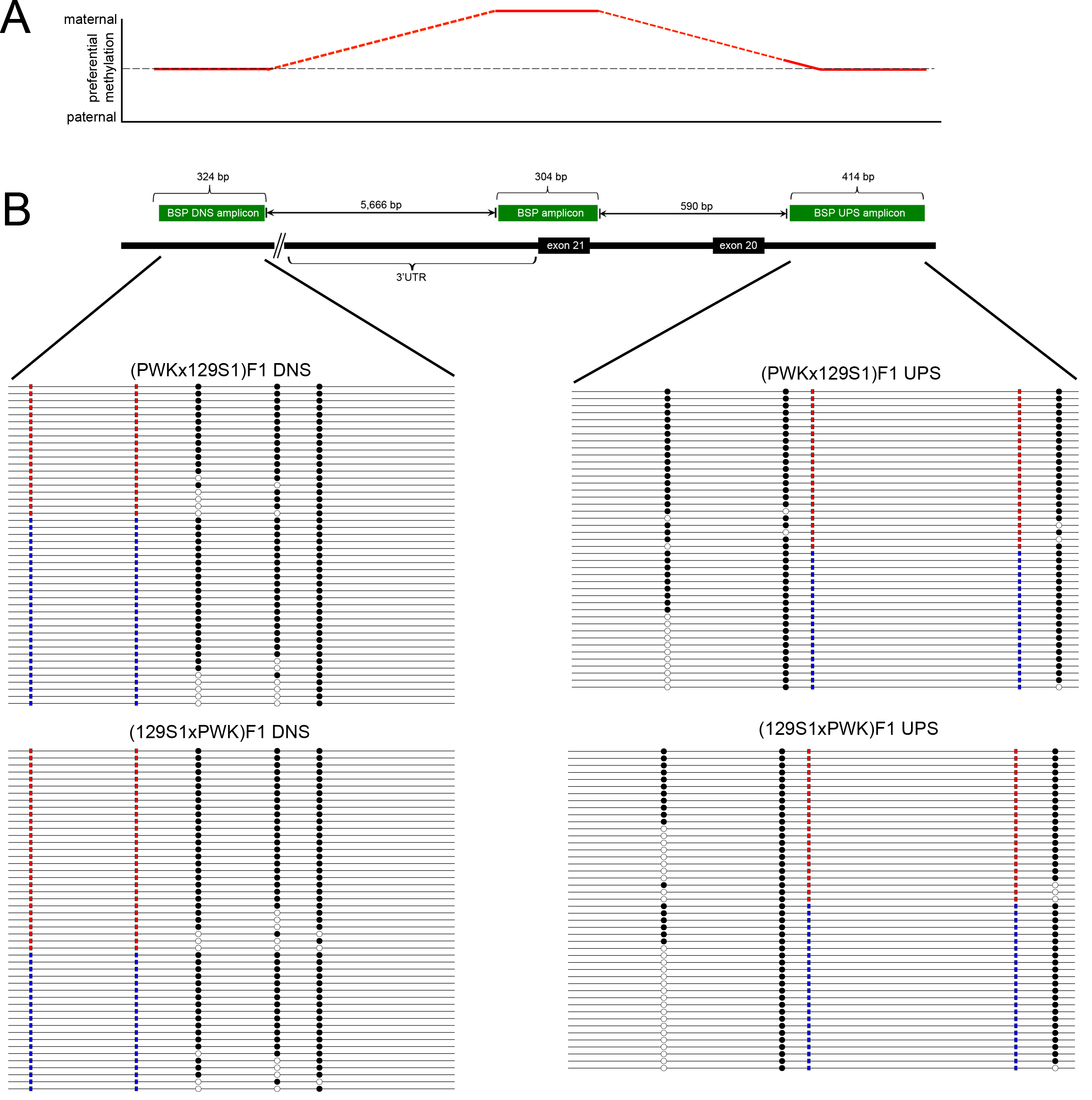

Supplement: Figure S5 — Bisulfite sequencing analysis of two regions flanking the Actn1 DMR in mouse liver tissues. Panel A shows regions of preferential methylation investigated by bisulfite sequencing. Solid red lines represent sequenced regions, while dotted lines represent gaps in sequenced regions. Panel B shows a schematic representation of the positions and sizes of the regions selected for methylation analysis by bisulfite sequencing respect to the location of the last two exons of Actn1 (exons 20 and 21, ENSMUSE00000114871 and ENSMUSE00000335764, respectively). Two regions, situated downstream (DNS BSP amplicon) and upstream (UPS BSP amplicon) of the region in which we observed differential methylation (BSP amplicon) (Figure 4), were selected for bisulfite sequencing analysis and the results are shown below the schematic. Each horizontal line represents a unique clone. Red and blue marks symbolize maternal and paternal alleles, respectively, of strain-specific variants. Open circles represent unmethylated CpGs, while closed circles are methylated CpGs. (TIF) [file pone.0048936.s005.tif]

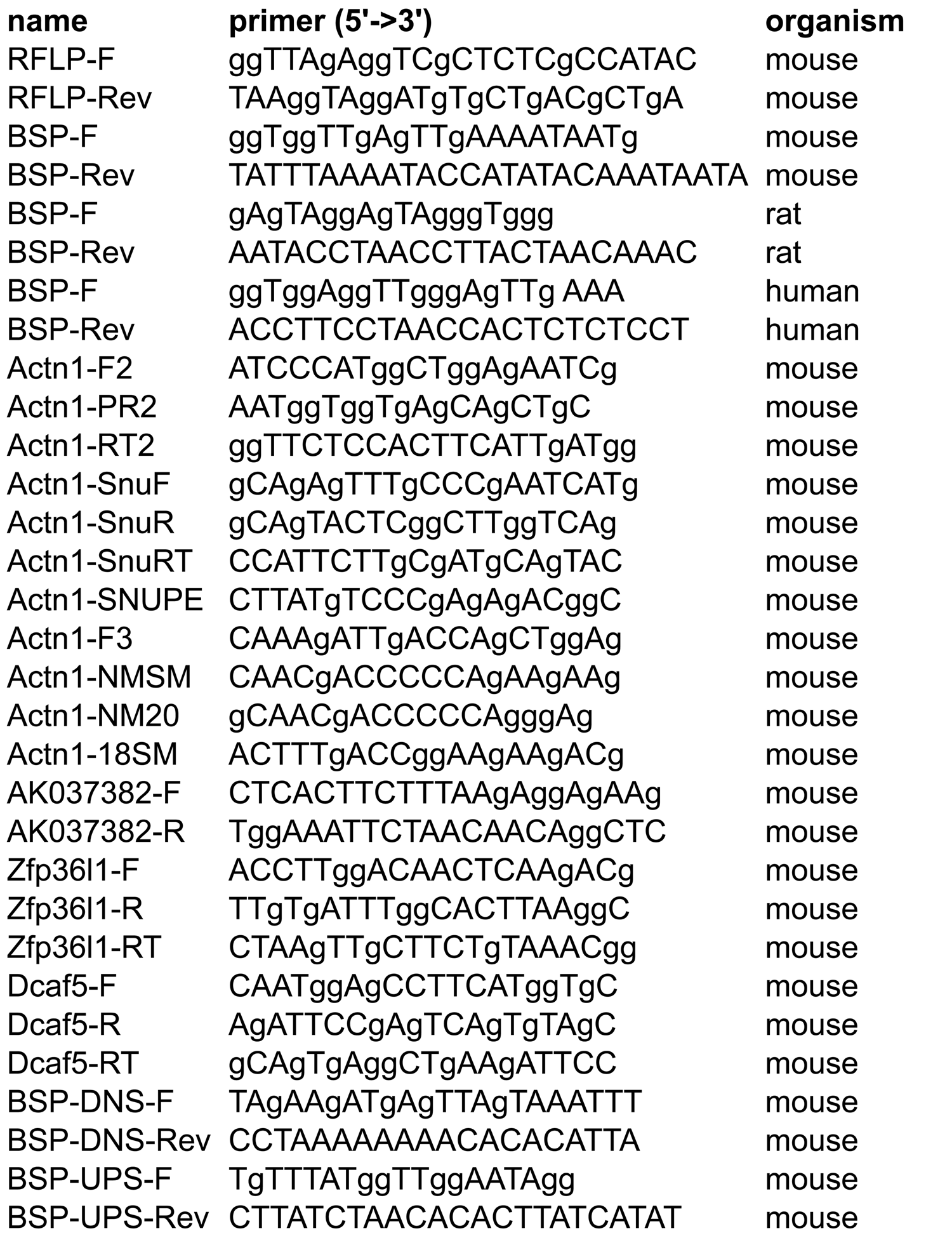

Supplement: Table S1 — List of primers used in the MS_RFLP (RFLP-), Bisulfite-PCR (BSP-), RT-PCR and sequencing or SNuPE (Snu-) analyses. (TIF) [file pone.0048936.s006.tif]

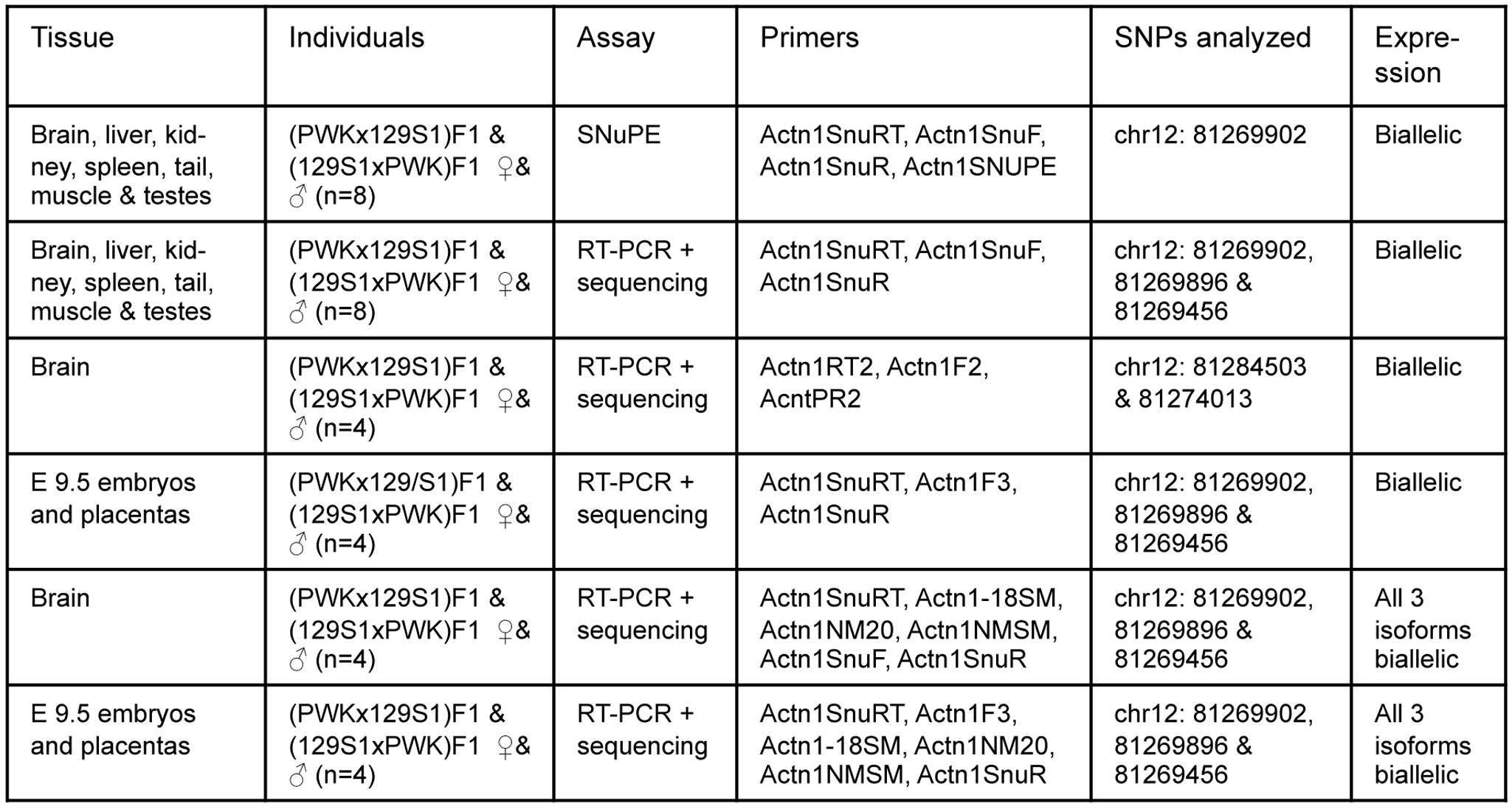

Supplement: Table S2 — Summary of Actn1 allelic expression analyses performed (see Supplemental Table S1 for primer’s sequences) (TIF) [file pone.0048936.s007.tif]

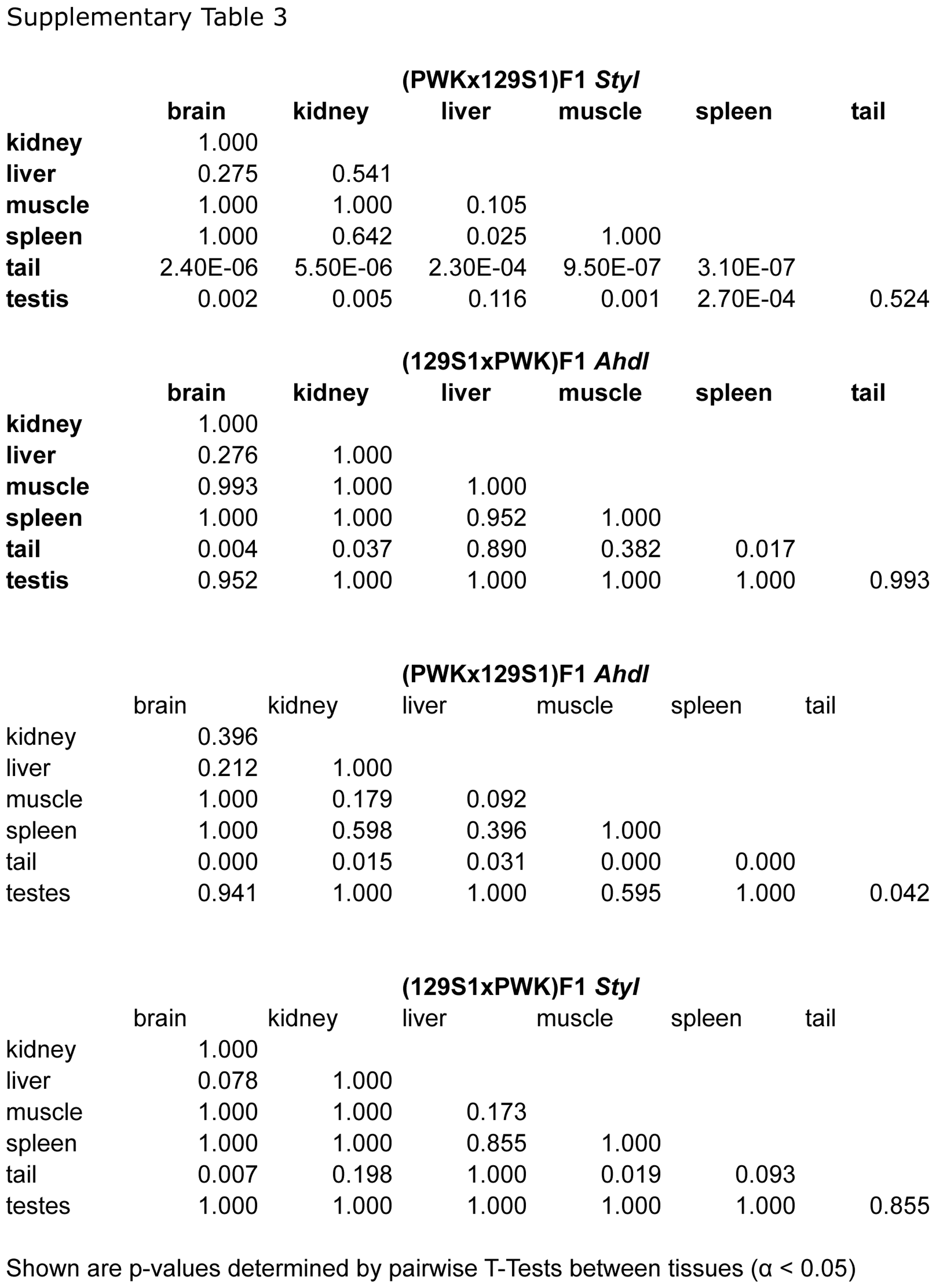

Supplement: Table S3 — Pairwise t-tests of percent maternal methylation at the Actn1 DMR between tissues. Shown are the p-values (α<0.05) (TIF) [file pone.0048936.s008.tif]
